# Comparing measured and reported change in gastrointestinal symptoms after initiation of metformin treatment: a questionnaire validation study

**DOI:** 10.1080/02813432.2025.2592696

**Published:** 2025-11-24

**Authors:** Peder af Geijerstam, Marika Wenemark, Bledar Daka, Stefan Jansson, Kenny Kalin, Olov Rolandsson, Karin Rådholm

**Affiliations:** ^a^ Department of Health, Medicine and Caring Sciences, Linköping University, Linköping, Sweden; ^b^ Primary Healthcare Center Cityhälsan Centrum, Region Östergötland, Norrköping, Sweden; ^c^ Department of Women’s and Children’s Health, Karolinska Institutet, Stockholm, Sweden; ^d^ School of Public Health and Community Medicine, Institute of Medicine, Sahlgrenska Academy, University of Gothenburg, Gothenburg, Sweden; ^e^ School of Medical Sciences, University Health Care Research Centre, Örebro University, Örebro, Sweden and Department of Public Health and Caring Sciences, Uppsala University, Uppsala, Sweden; ^f^ Department of Public Health and Clinical Medicine, Family Medicine, Umeå University, Umeå, Sweden; ^g^ Primary Healthcare Center Kärna, Region Östergötland, Linköping, Sweden; ^h^ The George Institute for Global Health, University of New South Wales, Sydney, Australia

**Keywords:** Treatment side-effects, bowel symptoms, primary health care, metformin, questionnaire, patient-reported outcome measure (PROM)

## Abstract

**Background:**

The majority of individuals in Sweden with type 2 diabetes have their sole health care provider in primary health care. Metformin treatment often causes gastrointestinal side-effects. Our aim was to construct and validate a questionnaire assessing gastrointestinal symptoms before and after starting metformin treatment for type 2 diabetes.

**Methods:**

In the Interaction Between Metformin and Microbiota (MEMO) study, 54 participants rated six gastrointestinal symptoms at baseline and after 2 months of metformin treatment in a questionnaire (measured change, i.e. the difference between assessments at these two time points), as well as direct assessment of perceived change in symptoms after 2 months in a separate validation questionnaire (reported change, i.e. how participants themselves have perceived the change between the same two time points). Spearman’s ρ was calculated and reported with its 95% CI.

**Results:**

The agreement between reported and measured change of symptoms, measured as Spearman’s ρ, was above 0.4 for 4 out of 6 symptoms (poor appetite 0.60 [95% CI 0.39–0.75], loose stool or diarrhea 0.58 [95% CI 0.37–0.74], flatulence 0.45 [95% CI 0.21–0.64], and abdominal pain 0.45 [95% CI 0.20–0.65]). The agreement was lower for nausea and vomiting, although these were numerically above 75% in agreement, likely due to few symptomatic participants overall.

**Conclusion:**

For common side-effect symptoms associated with metformin treatment, our study shows that symptom change measured as the difference between assessments at two different time-points was in overall agreement, validating the usability of the constructed questionnaire for metformin side-effects.

## 1. Introduction

The diabetes prevalence in Sweden is currently 6.0% and the absolute majority, 99% (*n* = 435 163) of individuals with type 2 diabetes are cared for in primary care [1]. In Denmark and Norway, the prevalence in 2021 was less than 4.0% [2]. Metformin is the first-line oral treatment of type 2 diabetes. However, gastrointestinal side-effects are common, and limit the use of metformin for some patients, and up to 30% of patients discontinue treatment or are unable to reach optimal therapeutic dosage due to side effects [3,4]. Diarrhea and nausea are the two most common gastrointestinal side effects, but others include flatulence and abdominal pain [3,4]. Considering these gastrointestinal side effects when choosing antidiabetic medications is important for patients in terms of quality of life and medication compliance [5]. The cause of gastrointestinal intolerance for metformin is unknown, but theories include loss-of-function of organic cation transporter 1, effects on intestinal glucose and bile acid metabolism, as well as effects on the gut microbiome [3,4].

In constructing questionnaires, several aspects of validity and reliability need to be considered, including construct validity [6,7]. The latter includes convergent validity, which measures the correlation between assessments that are expected to correlate [6,7].

Symptom change can be estimated in questionnaires directly by individuals reporting the change themselves (reported change or transition ratings), indirectly by individuals assessing current symptoms at different time points, after which the differences between those assessments are calculated (measured change), and by the ‘then-test’ method through which individuals rate absolute symptoms both current and retrospectively [8] (Box 1).

Box 1. Estimation of symptom change in questionnaires.

**Measured change**: Participants assess their current symptoms at the beginning and end of the reference period, and the difference between the two assessments is used as measured change;

**Reported change**: Participants assess the change of symptoms at the end of the reference period, and their direct assessment of change is used as reported change.

**Then-test method**: Individuals rate their current symptoms, as well as their symptoms in retrospect.

These three methods have different strengths and weaknesses and may yield different estimates of the changes. For direct transition ratings respondents need to be able to compare the current status to the situation at a given earlier time point [8]. This can be difficult if the respondent is supposed to relate to the situation, for example, 12 months ago but may be easier when it is a comparison between well-defined time periods such as before and after getting a disease or starting a new treatment [9]. For comparing current symptoms at different time points (measured change), there is a risk that respondents use the highest response option at baseline for daily pain and that it will not be possible to detect worsening of symptoms. In such a situation, there is a risk that the measured change does not capture the respondent’s experience [10].

Surprisingly few previous studies can be found describing and comparing these different ways to measure change in symptoms or functions, despite the importance and the wide use of questionnaires to evaluate symptoms in research and clinical practice. Of questionnaires for gastrointestinal symptoms, many do not discuss the difference between reported and measured change, and many questionnaires of medication side-effects ask participants to rate symptoms at one occasion and indicate if they think they are caused by the medication, rather than to ask participants if symptoms have changed after commencement of the drug [11–14]. Also, direct transition ratings may be based on other time frames than those intended, or without actual comparison to past states at all [9]. In addition, respondents may compare their current symptoms with, for example those at the time of diagnosis rather than the commencement of treatment [15].

In preparation of the Interaction Between Metformin and Microbiota (MEMO) study, we found that there is no existing, validated questionnaire for individuals to assess the change in gastrointestinal symptoms that are commonly attributed to metformin treatment. Thus, the aim of this study was to construct and validate a questionnaire on these gastrointestinal side-effects, and specifically, the assessment of change in bowel symptoms over time. Our hypothesis was that measured change correlates to reported change and thus reflects the change also from the patient’s perspective in terms of their direct experiences of the change.

## 2. Materials and methods

The aim was to develop a relatively brief questionnaire. An expert group with extensive knowledge of the target patient population reviewed existing, more comprehensive questionnaires. A survey methodologist specialized in questionnaire development (MW) was consulted. The symptoms used in the questionnaire were informed by the most common gastrointestinal side effects of metformin treatment [16].

The constructed questionnaire was first validated in terms of language, relevance and stakeholder engagement by using a simplified version of cognitive interviews with 7 patients to ensure that questions were easy to understand and answer, from the respondents’ perspectives. The interviews were conducted by one of the researchers (OR) and a GP registrar at two primary health care centers. The patients were asked to ‘think aloud’ when answering the questionnaire and were asked to comment on any ambiguities [17].

For the construct validity, we aimed at a sample size of at least 50 participants and thus an item-response ratio of well above 1:3 [18]. The data were collected from February 20 through December 31 of 2019 in Västerbotten County, Sweden. The 54 participants, newly diagnosed with type 2 diabetes, were recruited by their general practitioners during regular consultations, and the questionnaires were answered in Swedish by paper and pencil. In the study, participants were asked to assess 6 possible, previously well described metformin gastrointestinal side-effect symptoms (poor appetite, nausea, vomiting, loose stool or diarrhea, flatulence, and abdominal pain) at two time points.

The first questionnaire was administered twice: at baseline (i.e. before starting metformin treatment) and after 2 months of metformin treatment (hereafter referred to as the MEMO study questionnaire). Furthermore, at the time of the 2-month follow-up an additional validation questionnaire was used in which they were asked to report on the direct change in symptoms after they started with the treatment (hereafter referred to as the validation questionnaire). In the MEMO study questionnaire, symptoms were assessed by a unipolar ordinal scale: ‘no symptoms’, ‘symptoms at one or a few instances’, ‘symptoms daily’ or ‘symptoms several times daily’. The assessments at follow-up were compared to the assessments at baseline resulting in changes of symptoms graded as ‘much worse’, ‘somewhat worse’, ‘unchanged’, ‘somewhat better’, and ‘much better’ for each item. ‘Somewhat’ indicated one step difference on the ordinal scale and ‘much’ indicated at least two steps difference (e.g. going from ‘no symptoms’ at baseline to ‘symptoms daily’ or ‘symptoms several times daily’ was graded as ‘much worse’). These results will be referred to as the measured change.

In the validation questionnaire, the direct changes of the same symptoms were assessed on a bipolar ordinal scale; ‘much worse’, ‘somewhat worse’, ‘unchanged’, ‘somewhat better’ and ‘much better’. These results will be referred to as the reported change.

The reported change was compared to measured change and the differences were categorized as ‘2 steps more negative’, ‘1 step more negative’, ‘in agreement’, ‘1 step more positive’ or ‘2 steps more positive’ in reported change.

Rated worsening symptoms in measured change were tabulated with the number and percentages of rated worsening symptoms in reported change. Ad hoc, the results of the symptom assessments of flatulence and vomiting, respectively, were also cross tabulated in detail to allow analysis of these results. Numerical agreement as well as Spearman’s rank correlation coefficient ρ and its 95% CI between measured and reported change was calculated. Correlation coefficients were categorized as <0.4, 0.4 to <0.6, and ≥0.6, and correspondingly labelled as insufficient, moderate, or good, respectively. Missing data was handled by listwise deletion.

R version 4.3.1 (R Core Team, Vienna, Austria) and RStudio version 2023.09.0 + 463 (Posit Software, Boston, MA, USA) were used for data analysis.

## 3. Ethics approval and consent to participate

The MEMO study was approved by the Regional Ethical Review board in Umeå (2019-193-31 M) and adheres to the Declaration of Helsinki. All participants gave written, informed consent to participate.

## 4. Results

Of 59 participants, 54 (92%) answered both the MEMO study questionnaires at baseline and follow-up and the validation questionnaire at the 2-month follow-up for at least one symptom assessment, and 50 (85%) for all the symptom assessments. Reasons for non-participation have not been evaluated. Both partial and complete responses were included in our analysis, thus resulting in 54 included participants. Of those, 31 (57%) were men and the median age was 64 (Q1–Q3 61–70) years. Amongst participants, the daily dose of metformin was 500 mg for 21 (39%), 1000 mg for 24 (44%), 1500 mg for 2 (4%), and 2000 mg for 3 (6%) participants. Of these, one participant using 500 mg of metformin discontinued treatment during the initial 2-month period due to itching.

**Figure 1.**
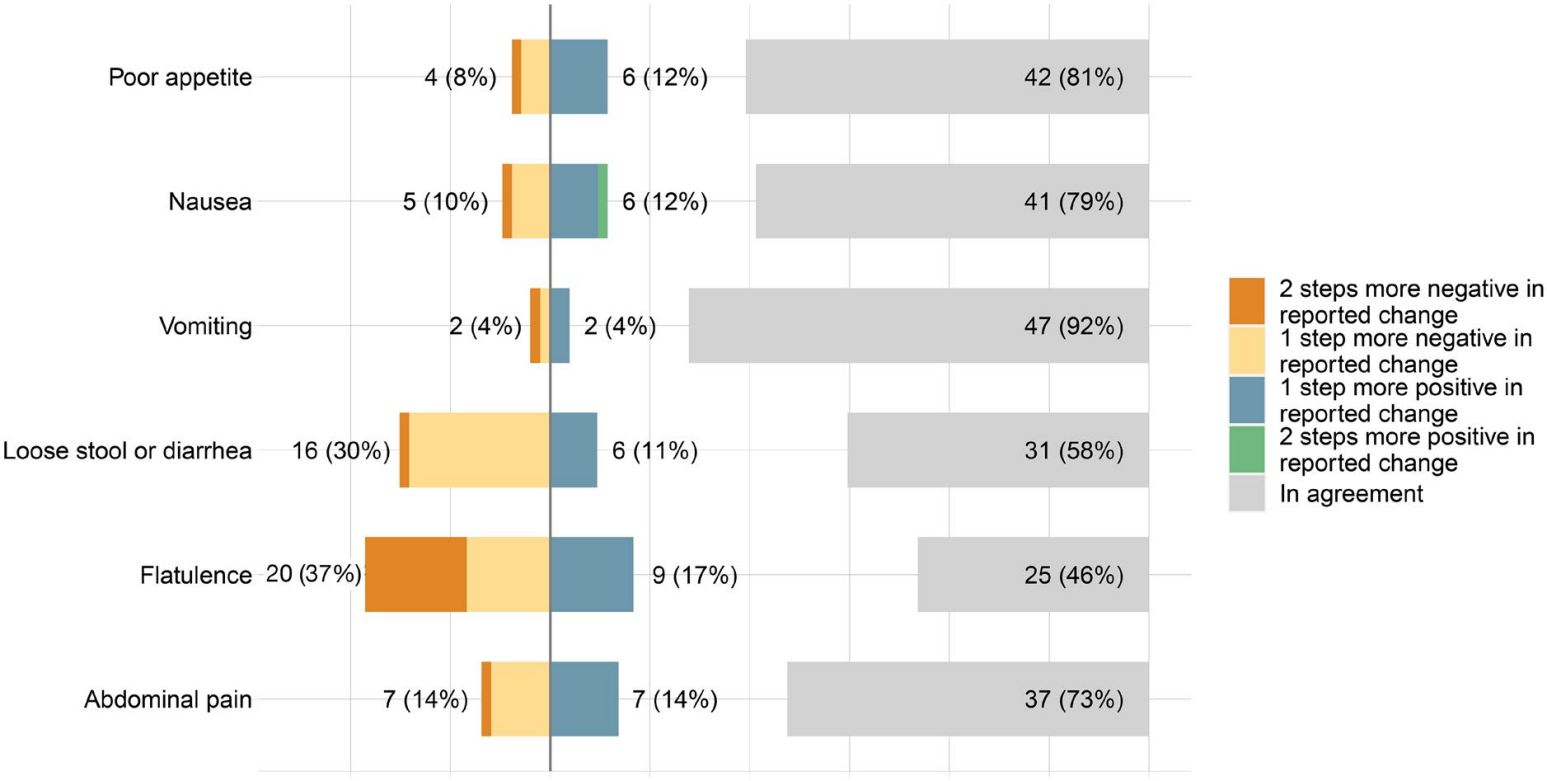
A scale chart showing the number and proportions (%) of each symptom in agreement (shown separately in light grey to the right), and 1 or 2 steps more positive (in blue and green) and negative (in yellow and orange) respectively, when comparing measured vs reported change.

The numeric agreement between reported and measured symptom changes was ≥72% for poor appetite, nausea, vomiting, and abdominal pain, with the highest agreement for vomiting, with 92% of respondents, Table 1, and Figure 1, as well as Supplementary Table 1 (all the assessments). For flatulence and loose stool or diarrhea the numeric agreement was lower (46% and 58%) and for both symptoms the reported change was more negative than the measured change. In total, the number of respondents with a discrepancy of symptoms ratings of at least 2 steps between measured and reported change was very low (2-4%) for all symptoms except for flatulence, where 11 (20%) rated reported change of at least 2 steps differently from measured change, Table 1.

**Table 1.** The agreement between reported and measured change of symptoms is shown as numbers and proportions (%).

	Difference between reported vs measured symptom change rating				Spearman’s correlation coefficient, ρ (95% CI)	Missing, n
	In agreement	At least 1 step more negative	At least 1 step more positive	At least 2 steps difference		
Poor appetite	42 (81%)	4 (8%)	6 (12%)	1 (2%)	0.60 (0.39 to 0.75)	2
Nausea	41 (79%)	5 (10%)	6 (12%)	2 (4%)	0.23 (−0.05 to 0.47)	2
Vomiting	47 (92%)	2 (4%)	2 (4%)	1 (2%)	−0.01 (−0.29 to 0.26)	3
Loose stool or diarrhea	31 (58%)	16 (30%)	6 (11%)	1 (2%)	0.58 (0.37 to 0.74)	1
Flatulence	25 (46%)	20 (37%)	9 (17%)	11 (20%)	0.45 (0.21 to 0.64)	0
Abdominal pain	37 (73%)	7 (14%)	7 (14%)	1 (2%)	0.45 (0.20 to 0.65)	3

Proportions are calculated from all valid responses (excluding missing). In agreement shows the number of participants for which the symptom change was the same in reported vs measured change. At least 1 step more negative and positive shows the number of participants for which the symptom change was 1 step more negative and positive respectively for reported versus measured change. At least 2 steps difference shows the number of participants for which the symptom change was at least 2 steps different (either more negative or positive) in reported vs measured change.

Of respondents, 36 (67%) rated worsening symptoms according to measured change for at least one of the symptoms. Between 1–5 (2-9%) of respondents that did not rate worsening of symptoms according to measured change, rated worsening symptoms in reported change, except for flatulence for which 10 (19%) of respondents rated worsening of symptoms. For all symptoms, few respondents (2-4%) indicated much worse symptoms in reported change, Table 2.

**Table 2.** Worsening of symptoms in measured change, as well as the differences between worsening symptoms as identified by the two ways of measuring symptom change.

	Worsening symptoms by measured change	Worsening symptoms by measured change but not by reported change	Worsening symptoms by reported change – but not by measured change	Much worsening symptoms by reported change but not by measured change
Poor appetite	10 (19%)	4 (8%)	1 (2%)	1 (2%)
Nausea	7 (13%)	5 (10%)	3 (6%)	1 (2%)
Vomiting	2 (4%)	2 (4%)	1 (2%)	1 (2%)
Loose stool or diarrhea	13 (25%)	5 (9%)	5 (9%)	0 (0%)
Flatulence	18 (33%)	5 (9%)	10 (19%)	2 (4%)
Abdominal pain	11 (21%)	6 (12%)	3 (6%)	0 (0%)

n (% of all responses).

**Figure 2.**
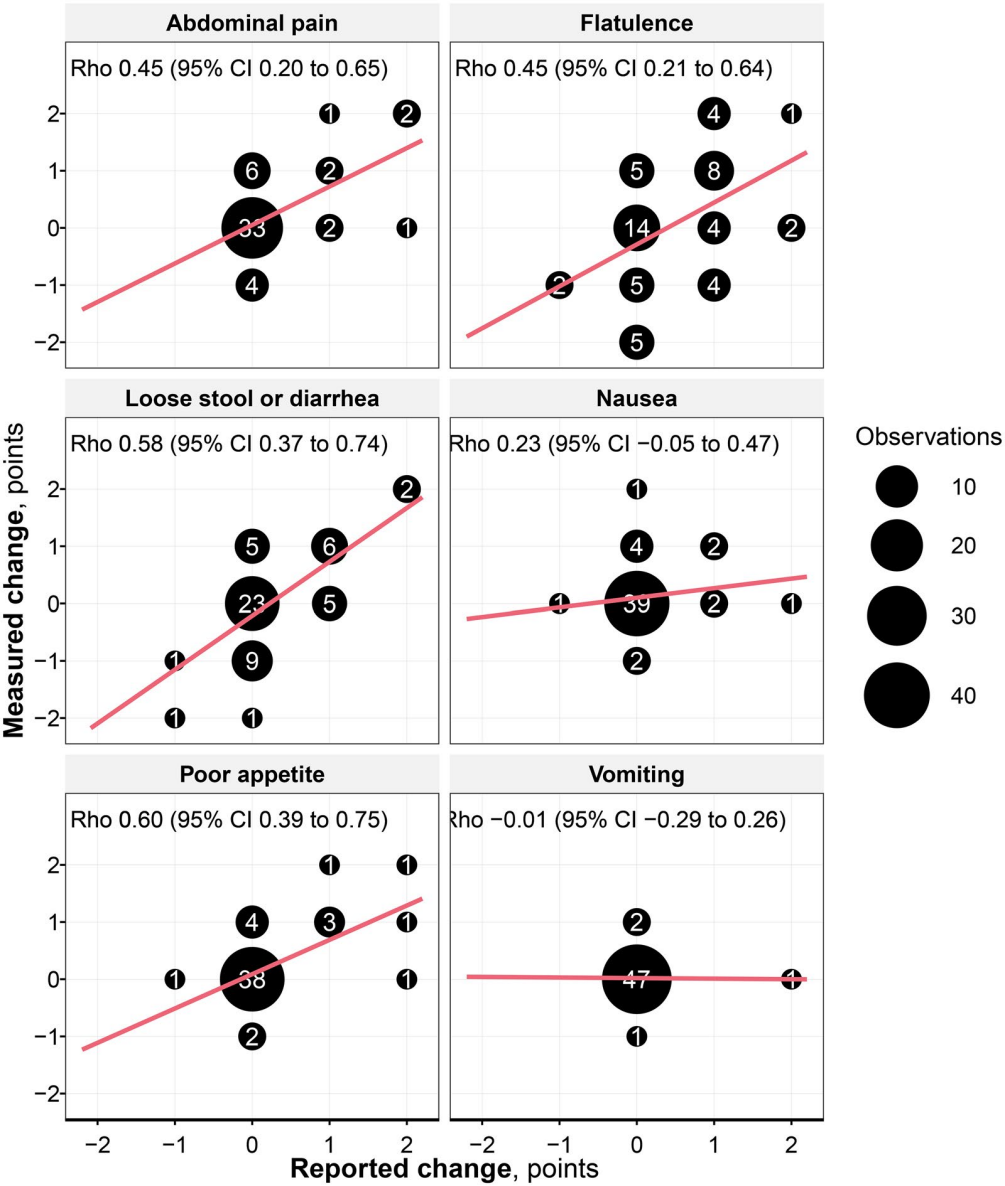
A scatter plot of observations with measured change on the y-axis and reported change on the x-axis, with the size corresponding to the number of observations (written in white), and the Spearman’s rank correlation coefficient.

Spearman’s ρ (95% CI) for poor appetite, loose stool or diarrhea, flatulence, and abdominal pain was between 0.45 and 0.60, Table 1 and Figure 2. For both nausea and vomiting, Spearman’s ρ (95% CI) was less than 0.4. When comparing women and men, the largest difference was seen for flatulence, where women showed a good correlation with Spearman’s *r* = 0.64 (0.18–0.84), but 32% of men had >1 step difference in reported change, with Spearman’s *r* = 0.29 (−0.07 to 0.59).

When analyzing flatulence assessments in detail, 8 (14.8%) indicated worsening symptoms in reported change and no change in symptoms between baseline and at 2 months (measured change). Of these, 2 (3.7%) reported much worse symptoms in reported change, Supplementary Table 2. For the measured vs reported change of vomiting assessments, 46 (90%) of participants assessed no issues neither at baseline nor follow-up of measured change, and unchanged symptoms of reported change, Supplementary Table 3.

## 5. Discussion

In this study comparing reported and measured assessments of change in bowel symptoms during the first 2 months of metformin treatment in 54 individuals, we found that the measured change was overall in alignment with the reported change for possible gastrointestinal metformin side-effects. Of respondents, 36 (67%) were identified as having one or more gastrointestinal symptoms. For poor appetite, nausea, vomiting, loose stool or diarrhea, and abdominal pain, respondents that indicated worse symptoms in reported change but no worsening in measured change were at most 9%, and much worse symptoms in reported change but no worsening in measured were at most 2%. Nausea and vomiting had a Spearman’s ρ less than 0.4, indicating an insufficient correlation. However, this may be explained by the high number of asymptomatic participants. It is therefore difficult to draw any conclusions for nausea and vomiting based on our study sample.

Although the overall number of participants with gastrointestinal symptoms was high, few (4% or less, apart from flatulence) indicated a worsening of more than 1 step on the response scale between before metformin treatment was initiated and after two months on metformin. For flatulence, however, 19% and 4% of respondents indicated worse and much worse symptoms, respectively, in reported change alone. Thus, fewer respondents were numerically in agreement between the measured and reported change than for the other assessed symptoms, although Spearman’s ρ was acceptable. This numerical lack of agreement may be explained by the high prevalence of flatulence, which is physiological and common, often without an explanatory morbidity or medication [19]. Therefore, it could be argued that a symptom change corresponding to one step may simply correspond to physiological variation rather than a metformin side effect.

In a few cases in our study, such as the 2 respondents that rated flatulence ‘several times per day’ both at baseline and follow-up in measured change, while rating it as ‘much worse’ in reported change, a ceiling effect may have concealed worsening symptoms in measured change. Such ceiling effects must be considered in any questionnaire using measured change, although it appears to have had a limited influence in the current study questionnaires.

Some sex differences were observed, most notably for flatulence. However, given the small sample size and absence of an a priori hypothesis supporting a biological or behavioral explanation, these findings could be due to chance and should be interpreted with caution.

Previous studies comparing reported and measured change have often shown low to moderate, or highly variable, agreement between assessments [8]. Suggested explanations include difficulties to recollect past states, and an unequal influence of the present state with transition ratings thus biased to correlate with the present status [8,15,20,21]. Transition ratings may be based on other time frames than those intended, or without actual comparison to past states at all [9]. Transition ratings may also be influenced by response shift and recall-related biases, such as implicit theory and social desirability bias, affecting how respondents remember and interpret a symptom at a previous time [22,23]. However, response shift may also affect measured change [10]. Although response shift effects have been reported to vary between domains [23], and often have low effect sizes [10], they may be larger when measuring direct vs indirect change [8], and some studies have suggested that a combination of reported and measured change to be used, when possible [24]. Transition ratings may therefore be problematic for measuring change at multiple occasions since the time frame will change and make it difficult for respondents to compare the present state to a desired earlier point in time. When starting a new medication, it may therefore be easier for the respondents to relate their present status to a period before without the need to define the period as specific weeks or months, but rather ‘before medication’. However, questions that are expressed in that way could also influence respondents to expect a change related to the introduction of the medication.

In many studies and in clinical care, measured change is used as a standard to compare health status on different occasions [8]. This is also considered the standard in this study. However, such measurements need to have a response scale that is specific enough to capture relevant changes. From a patient’s perspective, it is important that the measured change reflects their experience. If patients experience a worsening of symptoms that cannot be detected in the measured change due to e.g. ceiling effects, inadequate or too few response options, such change may not be noticed. It is therefore important to evaluate if the measured change reflects the perspective of the patients as one part of the validation of the questionnaire.

As mentioned, a limitation of our study was the low number of respondents in relation to the scarcity of nausea and vomiting symptoms, which may have affected the ability to assess the alignment of the two assessments. Furthermore, although the median age of 64 years is similar to the national mean, the proportion of men (57%) was slightly lower than the nationwide 64% [1], and inclusion of only individuals with newly diagnosed T2D limits generalizability. Of the symptoms assessed in this study, nausea and vomiting are symptoms that previously have been found to be less common [25]. Also, the symptom scale may have different functions for symptoms that appear as distinct events, such as vomiting, compared with those that may be the result of several aspects, for instance number of times, character, and duration, such as flatulence. Finally, in most questionnaires, it is not possible to ask about several aspects of each symptom such as intensity, frequency, degree of discomfort, or influence on daily life. Previous studies have discussed the issue of which dimension of severity that are measured, and that it may affect the lack of agreement between measured and reported change [24]. The MEMO study questionnaire asked about the frequency of symptoms which may be highly adequate for distinct events such as vomiting. That may explain why less distinct symptoms such as flatulence can be experienced as worse, even if the frequency estimations at the two time points are the same. Some participants with worsening symptoms may be missed in measured change because of this, even if that mostly concerns ‘somewhat worse’ symptoms according to reported change. “Much worse” has a high agreement for all symptoms. Future studies that include cognitive interviews could give a deeper understanding of the why and how the different assessments vary for different types of symptoms to expand the knowledge about how to best assess symptom changes. Finally, we studied relatively healthy individuals with type 2 diabetes, and external validity beyond this group, including individuals treated with metformin on other indications, could not be determined.

In conclusion, our study showed that symptom change measured as the difference between assessments at two different time points two months apart was in reasonable agreement with reported change for the common side-effect symptoms associated with metformin treatment. The MEMO questionnaire was thus considered valid and could be helpful to researchers and clinicians wanting to assess gastrointestinal metformin side-effects.

## Supplementary Material

Supplementary file clean.docx
