# Supplementary material for: Comparing measured and reported change in gastrointestinal symptoms after initiation of metformin treatment: a questionnaire validation study
Source: Scand J Prim Health Care. 2025 Nov 24;44(1):1–9. doi: 10.1080/02813432.2025.2592696 (PMC12918315; doi:10.1080/02813432.2025.2592696)
Supplement: Supplementary file clean.docx [file IPRI_A_2592696_SM3929.docx]

**Title:** **Comparing measured and reported change in gastrointestinal symptoms after initiation of metformin treatment: a questionnaire validation study**

Supplementary tables

**Supplementary table 1.** Change of symptoms shown as the numbers and percentages, with measured change shown in the rows and reported change shown in the columns. Assessments that agreed between measured and reported change are shaded in gray.

|  | Reported change | | | | |
| --- | --- | --- | --- | --- | --- |
| Measured change | Much worse | Somewhat worse | Unchanged | Somewhat better | Much better |
| Poor appetite | | | | | |
| Much worse | 1 (1.9%) | 1 (1.9%) | 0 (0.0%) | 0 (0.0%) | 0 (0.0%) |
| Somewhat worse | 1 (1.9%) | 3 (5.8%) | 4 (7.7%) | 0 (0.0%) | 0 (0.0%) |
| Unchanged | 1 (1.9%) | 0 (0.0%) | 38 (73.1%) | 1 (1.9%) | 0 (0.0%) |
| Somewhat better | 0 (0.0%) | 0 (0.0%) | 2 (3.8%) | 0 (0.0%) | 0 (0.0%) |
| Much better | 0 (0.0%) | 0 (0.0%) | 0 (0.0%) | 0 (0.0%) | 0 (0.0%) |
| Nausea | | | | | |
| Much worse | 0 (0.0%) | 0 (0.0%) | 1 (1.9%) | 0 (0.0%) | 0 (0.0%) |
| Somewhat worse | 0 (0.0%) | 2 (3.8%) | 4 (7.7%) | 0 (0.0%) | 0 (0.0%) |
| Unchanged | 1 (1.9%) | 2 (3.8%) | 39 (75.0%) | 1 (1.9%) | 0 (0.0%) |
| Somewhat better | 0 (0.0%) | 0 (0.0%) | 2 (3.8%) | 0 (0.0%) | 0 (0.0%) |
| Much better | 0 (0.0%) | 0 (0.0%) | 0 (0.0%) | 0 (0.0%) | 0 (0.0%) |
| Vomiting | | | | | |
| Much worse | 0 (0.0%) | 0 (0.0%) | 0 (0.0%) | 0 (0.0%) | 0 (0.0%) |
| Somewhat worse | 0 (0.0%) | 0 (0.0%) | 2 (3.9%) | 0 (0.0%) | 0 (0.0%) |
| Unchanged | 1 (2.0%) | 0 (0.0%) | 47 (92.2%) | 0 (0.0%) | 0 (0.0%) |
| Somewhat better | 0 (0.0%) | 0 (0.0%) | 1 (2.0%) | 0 (0.0%) | 0 (0.0%) |
| Much better | 0 (0.0%) | 0 (0.0%) | 0 (0.0%) | 0 (0.0%) | 0 (0.0%) |
| Loose stool or diarrhea | | | | | |
| Much worse | 2 (3.8%)^*^ | 0 (0.0%) | 0 (0.0%) | 0 (0.0%) | 0 (0.0%) |
| Somewhat worse | 0 (0.0%) | 6 (11.3%) | 5 (9.4%) | 0 (0.0%) | 0 (0.0%) |
| Unchanged | 0 (0.0%) | 5 (9.4%) | 23 (43.4%) | 0 (0.0%) | 0 (0.0%) |
| Somewhat better | 0 (0.0%) | 0 (0.0%) | 9 (17.0%) | 1 (1.9%) | 0 (0.0%) |
| Much better | 0 (0.0%) | 0 (0.0%) | 1 (1.9%) | 1 (1.9%) | 0 (0.0%) |
| Flatulence | | | | | |
| Much worse | 1 (1.9%) | 4 (7.4%) | 0 (0.0%) | 0 (0.0%) | 0 (0.0%) |
| Somewhat worse | 0 (0.0%) | 8 (14.8%) | 5 (9.3%) | 0 (0.0%) | 0 (0.0%) |
| Unchanged | 2 (3.7%) | 4 (7.4%) | 14 (25.9%) | 0 (0.0%) | 0 (0.0%) |
| Somewhat better | 0 (0.0%) | 4 (7.4%) | 5 (9.3%) | 2 (3.7%) | 0 (0.0%) |
| Much better | 0 (0.0%) | 0 (0.0%) | 5 (9.3%) | 0 (0.0%) | 0 (0.0%) |
| Abdominal pain | | | | | |
| Much worse | 2 (3.9%) | 1 (2.0%) | 0 (0.0%) | 0 (0.0%) | 0 (0.0%) |
| Somewhat worse | 0 (0.0%) | 2 (3.9%) | 6 (11.8%) | 0 (0.0%) | 0 (0.0%) |
| Unchanged | 1 (2.0%) | 2 (3.9%) | 33 (64.7%) | 0 (0.0%) | 0 (0.0%) |
| Somewhat better | 0 (0.0%) | 0 (0.0%) | 4 (7.8%) | 0 (0.0%) | 0 (0.0%) |
| Much better | 0 (0.0%) | 0 (0.0%) | 0 (0.0%) | 0 (0.0%) | 0 (0.0%) |

* Of which 1 rated 3 steps worse in reported change.

**Supplementary table 2.** Detailed analysis of flatulence. Assessments that were in agreement between measured and reported change are shaded in gray.

| Measured change | | Reported change at 2 months | | | | |
| --- | --- | --- | --- | --- | --- | --- |
| Symptoms at baseline | **Symptoms at 2 months** | **Much worse** | **Somewhat worse** | **Unchanged** | **Somewhat better** | **Much better** |
| No issues | **No issues** | 0 (0%) | 0 (0%) | 7 (13%) | 0 (0%) | 0 (0%) |
|  | **At one/some instances** | 0 (0%) | 3 (5.6%) | 2 (3.7%) | 0 (0%) | 0 (0%) |
|  | **Every day** | 0 (0%) | 4 (7.4%) | 0 (0%) | 0 (0%) | 0 (0%) |
|  | **Several times every day** | 0 (0%) | 0 (0%) | 0 (0%) | 0 (0%) | 0 (0%) |
| At one/some instances | **No issues** | 0 (0%) | 0 (0%) | 4 (7.4%) | 0 (0%) | 0 (0%) |
|  | **At one/some instances** | 0 (0%) | 2 (3.7%)^*^ | 4 (7.4%) | 0 (0%) | 0 (0%) |
|  | **Every day** | 0 (0%) | 3 (5.6%) | 1 (1.9%) | 0 (0%) | 0 (0%) |
|  | **Several times every day** | 1 (1.9%) | 0 (0%) | 0 (0%) | 0 (0%) | 0 (0%) |
| Every day | **No issues** | 0 (0%) | 0 (0%) | 5 (9.3%) | 0 (0%) | 0 (0%) |
|  | **At one/some instances** | 0 (0%) | 1 (1.9%)^*^ | 1 (1.9%) | 1 (1.9%) | 0 (0%) |
|  | **Every day** | 0 (0%) | 2 (3.7%)^*^ | 3 (5.6%) | 0 (0%) | 0 (0%) |
|  | **Several times every day** | 0 (0%) | 2 (3.7%) | 2 (3.7%) | 0 (0%) | 0 (0%) |
| Several times every day | **No issues** | 0 (0%) | 0 (0%) | 0 (0%) | 0 (0%) | 0 (0%) |
|  | **At one/some instances** | 0 (0%) | 0 (0%) | 0 (0%) | 0 (0%) | 0 (0%) |
|  | **Every day** | 0 (0%) | 3 (5.6%)^*^ | 0 (0%) | 1 (1.9%) | 0 (0%) |
|  | **Several times every day** | 2 (3.7%)^*,†^ | 0 (0%) | 0 (0%) | 0 (0%) | 0 (0%) |

* Assessments that are worse in reported change, but unchanged or better in measured change, representing worsened symptoms that could potentially be missed if using only reported change.

† Assessments that are at the maximum frequency both at baseline and follow-up in measured change, representing a potential ceiling effect.

**Supplementary table 3.** Detailed analysis of vomiting with columns showing the reported change, and rows showing measured change detailed as the baseline and 2-month assessments. Assessments that are worse in reported change, but unchanged or better in measured change, are indicated (*), representing worsened symptoms that could potentially be missed if using only reported change. Assessments that were in agreement between measured and reported change are shaded in gray.

|  |  | Reported change at 2 months | | | | |
| --- | --- | --- | --- | --- | --- | --- |
| Symptoms at baseline | **Symptoms at 2 months** | **Much worse** | **Somewhat worse** | **Unchanged** | **Somewhat better** | **Much better** |
| No issues | **No issues** | 1 (2%)^*^ | 0 (0%) | 46 (90%) | 0 (0%) | 0 (0%) |
|  | **At one/some instances** | 0 (0%) | 0 (0%) | 2 (4%) | 0 (0%) | 0 (0%) |
|  | **Every day** | 0 (0%) | 0 (0%) | 0 (0%) | 0 (0%) | 0 (0%) |
|  | **Several times every day** | 0 (0%) | 0 (0%) | 0 (0%) | 0 (0%) | 0 (0%) |
| At one/some instances | **No issues** | 0 (0%) | 0 (0%) | 0 (0%) | 0 (0%) | 0 (0%) |
|  | **At one/some instances** | 0 (0%) | 0 (0%) | 1 (2%) | 0 (0%) | 0 (0%) |
|  | **Every day** | 0 (0%) | 0 (0%) | 0 (0%) | 0 (0%) | 0 (0%) |
|  | **Several times every day** | 0 (0%) | 0 (0%) | 0 (0%) | 0 (0%) | 0 (0%) |
| Every day | **No issues** | 0 (0%) | 0 (0%) | 0 (0%) | 0 (0%) | 0 (0%) |
|  | **At one/some instances** | 0 (0%) | 0 (0%) | 1 (2%) | 0 (0%) | 0 (0%) |
|  | **Every day** | 0 (0%) | 0 (0%) | 0 (0%) | 0 (0%) | 0 (0%) |
|  | **Several times every day** | 0 (0%) | 0 (0%) | 0 (0%) | 0 (0%) | 0 (0%) |
| Several times every day | **No issues** | 0 (0%) | 0 (0%) | 0 (0%) | 0 (0%) | 0 (0%) |
|  | **At one/some instances** | 0 (0%) | 0 (0%) | 0 (0%) | 0 (0%) | 0 (0%) |
|  | **Every day** | 0 (0%) | 0 (0%) | 0 (0%) | 0 (0%) | 0 (0%) |
|  | **Several times every day** | 0 (0%) | 0 (0%) | 0 (0%) | 0 (0%) | 0 (0%) |

* Assessments that are worse in reported change, but unchanged or better in measured change, representing worsened symptoms that could potentially be missed if using only reported change.
